# Analysis of Casual Relationships between Social Determinants of Health in Iran: Using Fuzzy Cognitive Map

**DOI:** 10.34172/jrhs.2022.101

**Published:** 2022-12-29

**Authors:** Nafiseh Salehnia, Abbas Assari Arani, Alireza Olyaeemanesh, Hossein Sadeghi Saghdel

**Affiliations:** ^1^Department of Economic Development and Planning, Faculty of Management and Economics, Tarbiat Modares University, Tehran, Iran; ^2^Health Equity Research Center (HERC), Tehran University of Medical Sciences, Tehran, Iran; ^3^Department of Health Economics, National Institute of Health Research, Tehran University of Medical Sciences, Tehran, Iran

**Keywords:** Fuzzy cognitive map, Health determinants, Health policy, Public health

## Abstract

**Background:** Health is a fundamental issue in recent years, highlighting the importance of harmonizing the policies of any sector with health strategies. The present study aims to identify the socio-economic factors affecting health and to provide a cognitive map of the social determinants of health in Iran.

**Study Design:** A retrospective cohort study.

**Methods:** This study follows a developmental process with an exploratory sequential mixed methods approach. First, a meta-synthesis qualitative method determines the most critical health determinants by reviewing 54 studies from 2000 to 2019. Then, the fuzzy cognitive map (FCM) is drawn based on interviews with six experts to derive the causal relationships among the social determinants of population health in Iran.

**Results:** Stage 1 introduces 170 factors as the health determinants, classified into four levels: individual, local, national, and global levels, with 7, 4, 13, and 3 branches, respectively. According to the causal relationships and the out-degree (od) index, the most effective factors are the economic system (18.24), governance and policy-making (17.13), and national policies (16.93). According to the degree of centrality, these factors are the economic system (33.27), health system (30.37), and governance and policy-making (30.15).

**Conclusion:** Considering health as a complex and comprehensive system, the resulting FCM displays that the policies developed in other sectors than health are profoundly affecting population’s health in Iran. Specifically, the comparative analysis of this research shows that policies regarding the economic system and people’s livelihood are more effective than the policies regarding the health system itself on the population’s health in Iran.

## Background

 In addition to the positive effect of the healthcare sector on individuals’ health, socio-economic factors play an additional and significant role in the population’s health.^1^ According to the World Health Organization (WHO) report, 50% of inequalities in major non-communicable diseases are for social inequalities in risk factors. Furthermore, investments in the non-health sector cause 50% of the decrease in the mortality rate of children under 5 years old within 1990-2010. Integrated medical and social services indicate a 10-fold reduction in child mortality between the treatment and control groups.^2^ The concept of social determinants of health refers to conditions where people are born, grow, live, and age, and inequality in these conditions leads to inequality in people’s health.^3^ Many of these health determinants depend on the other sectors outside the health system, including immigration policies, laws and regulations, demographic, economic and political factors, criminal justice system, labor market system, institutions and agencies,^4^ economic stability, education, neighborhood and living environment,^5^ perceived neighborhood crime,^6^ adjustable housing, sustainable employment, access to healthy food and quality schools,^7^ economic system,^8^ and workplace conditions.^9^

 In a survey, 10% of the population’s health was determined by the physical environment, 20% by clinical healthcare (availability and equity), 30% by individual health behaviors, and 40% by economic and social factors. The socio-economic factors not only account for the largest part of health, but also affect the second factor, i.e., individual behaviors, to a large extent.^10^

 Public policies and decisions taken in all the government sectors and levels affect the society’s health and justice in health and the capacity of health systems to protect health and satisfy health needs.^11^ For example, a 10% decrease in the prices of fruits, vegetables, nuts, and grains would prevent 19 600 deaths per year. Adding a 30% subsidy to healthy foods can also result in the greatest decrease in mortality rates.^12^ “Health in All Policies” (HiAPs) approach considers the effects of other policies and laws on health via health determinants.^13^

 Another reason for the importance of paying attention to HiAP is that some seemingly unrelated policies are likely to create unwanted effects that are not measured and resolved. To achieve policy coherence in the government, the health sector should recognize the other sectors’ goals and develop a shared understanding of health, its determinants, and wider social well-being or quality of life.^14^

 Public health affects population health both directly and indirectly via social determinants.^15^ However, public health in Iran, has not been much helpful in addressing the social and economic factors determining health. A cognitive map of the social determinants of health is in line with the HiAP approach, which enables the government to act in an integrated way in responding to the society’s health. While there is a significant lag between political decisions and their impact on health outcomes, impacts on health determinants can be seen much earlier. Hence, evaluation of the effects of every policy and decision, whether big or small, personal or political, on the health determinants is necessary. Therefore, the main issue is to make other sectors aware of the effects of their decisions on health and to integrate health goals with other policies.

 This study aims to answer the following three main questions:

- What are the social determinants of population health in Iran?
- What is the importance of each social determinant of health in determining the health status in Iran?
- How do health determinants interact with each other in Iran?

## Materials and Methods

 This study designs a developmental process with an exploratory sequential mixed methods approach. In this approach, qualitative and quantitative steps can complement each other via discovery and verification.^16^

###  Step 1. Qualitative step: Meta-synthesis approach

 Regarding the first research question, the meta-synthesis approach^17^ identified the most important determinants of population health based on the studies conducted within 2000-2019. This stage searched for numerous references to select and review about 3000 articles related to the topic. Among them, this method selected 54 studies for analyzing and extracting the social determinants of health.

 The Kappa index measures and controls the quality of the findings. In this way, an associate researcher (one of the elites in the field of social determinants of health) attempted to assign codes in the form of concepts and components without becoming aware of the process of integration and initial classification of the concepts. Performing the calculations on the agreement and disagreement values, the value of the Kappa index was calculated to 0.96 (i.e., high-level agreement).^18^

###  Step 2. Quantitative step: FCMs

 Fuzzy cognitive maps (FCMs), proposed by Kosko in 1986, constitute an expert-based method of knowledge development in soft domains such as political and military sciences, history, international relations, and organization theory. Instead of using a binary indicator such as an arrow or no arrow to define the certainty of relationships in the map, fuzzy maps allow a range of weights allocated to the relationship (arrow). A fuzzy set is described by means of a membership function.^19^ This technique provides a visual representation of different knowledge using well-established analytical tools.^20^ The FCMs can successfully represent knowledge and experience, introducing concepts for the essential elements and through the use of cause and effect relationships among the concepts.^21^

 An FCM provides a causal graphical representation consisting of interrelated concepts. Fuzzy cognitive mapping draws each factor as a node and represents each relationship as an edge (arrow) linking nodes. The arrows represent assumptions about causal relationships based on data or unwritten knowledge.^22^

 Each FCM has a number of concepts. These concepts represent conceptual characteristics of the system, and weight W_ij_ denotes the cause-and-effect of one concept on another. In general, concepts of an FCM represent key factors and characteristics of the modeled complex system. Values of objects and interrelations range from 0 to 1. In mathematical terms, FCM is a vector of object values and a matrix of interrelation values.^19^

 The present study builds an FCM that models expert’s conceptualization of the factors influential in population health in Iran. Estimating the fuzzy weights needs to capture the opinions of domain experts about the strength of the effects of interconnected factors of the FCM.^23^ The essence of fuzzy logic is to allow experts to express their knowledge without being forced to use numerical values. For this reason, at the stage of estimating the values of interrelations in the FCM, model experts should use linguistic values and not associate them with any numerical counterparts. However, for further simulations, the researcher needs to operate on numbers that are obtained from linguistic values under the so-called “defuzzification” procedure.^19^ In this part, experts are required to choose the closest linguistic expression to their rationale in terms of linguistic expression. To assess interrelations, experts analyzed pairs of objects and determined if there was an influence between them, how strong it was, and whether it was positive or negative (“no influence”, “very low influence”, “low influence”, “medium influence”, “high influence”, “very high influence”, and influence close to 1).

 For transforming experts’ linguistic expressions into the numerical values (crisp weights) that define the FCM’s interrelations, this study utilized the modified weighted mean of maximum method.^19,23,24^ Eq.1 represents defuzzification.


(1) $$W=\frac{\sum_{i=1}^{N}O_{i}Z_{i}}{\sum_{i=1}^{N}O_{i}}$$

Where W is the crisp weight and N is the total number of experts participating in the questionnaire. O_i_ and Z_i_ are the maximum value of membership function corresponding to the linguistic value estimated by Expert i and defuzzified linguistic value estimated by Expert i, respectively. Assuming that influences were estimated without scaling, the indication of a linguistic value was associated with the maximum value of a corresponding membership function. As this value is equal to 1, the transformation procedure was based on calculating the mean of Z_i_ values corresponding to the assessments of 6 experts participating in the study. Calculating all strengths of influence values allowed for development of the interrelation matrix. In the final step, the experts reviewed the obtained results to avoid misleading data. Such spurious results occur when the estimations imply a significant relationship that is logically unrelated and lacks a theoretical foundation. The experts can easily identify and remove such counterfeit results.^25^

 After assigning degrees of intensity to the cause-and-effect relationships, FCMs were created using the FCMapper v1.1 and Pajek24 v 5.16 software packages. The metrics used to compare components and for structural analysis of FCMs are^26,27^:

 1) In-degree of each component (id):

Eq. 2 represents id or the cumulative strength of connections with which a component is influenced by other components.


(2) $$id_{i}=\sum_{k=1}\left|a_{ji}\right|$$

2) Out-degree of each component (od):

Eq. 3 shows od or the cumulative strength of connections with which a component influences other components.


(3) $$od_{i}=\sum_{k=1}\left|a_{ij}\right|$$

3) Degree of centrality (DoC):

 Indicates (*a*) the total influence (positive and negative) to be in the system or (*b*) the conceptual weight/importance of individual concepts.

Eq. 4 shows DoC or the cumulative strength of connections a component has (in and out). The higher the value, the greater is the importance of all concepts or the individual weight of a concept in the overall model.


(4) $$DoC_{i}=od_{ij}\text{+}id_{ji}$$

Where *a* represents each arrow, i is the transmitter node of arrow *a*, *j* signifies the receiving node of arrow.

## Results

**Figure 1:**
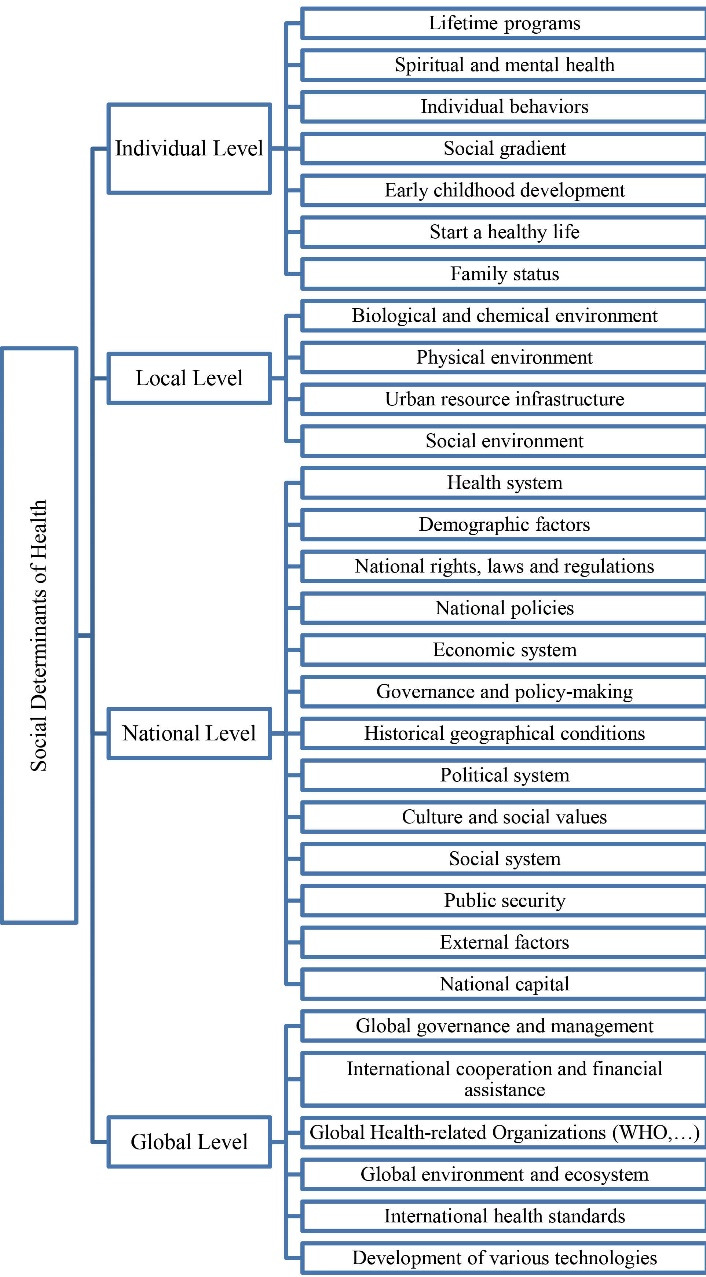
Social determinants of population health

 This section presents the resulting analysis of reviewing the selected references. Figure 1 displays the social determinants of health into four levels: individual, local, national, and global.

**Figure 2:**
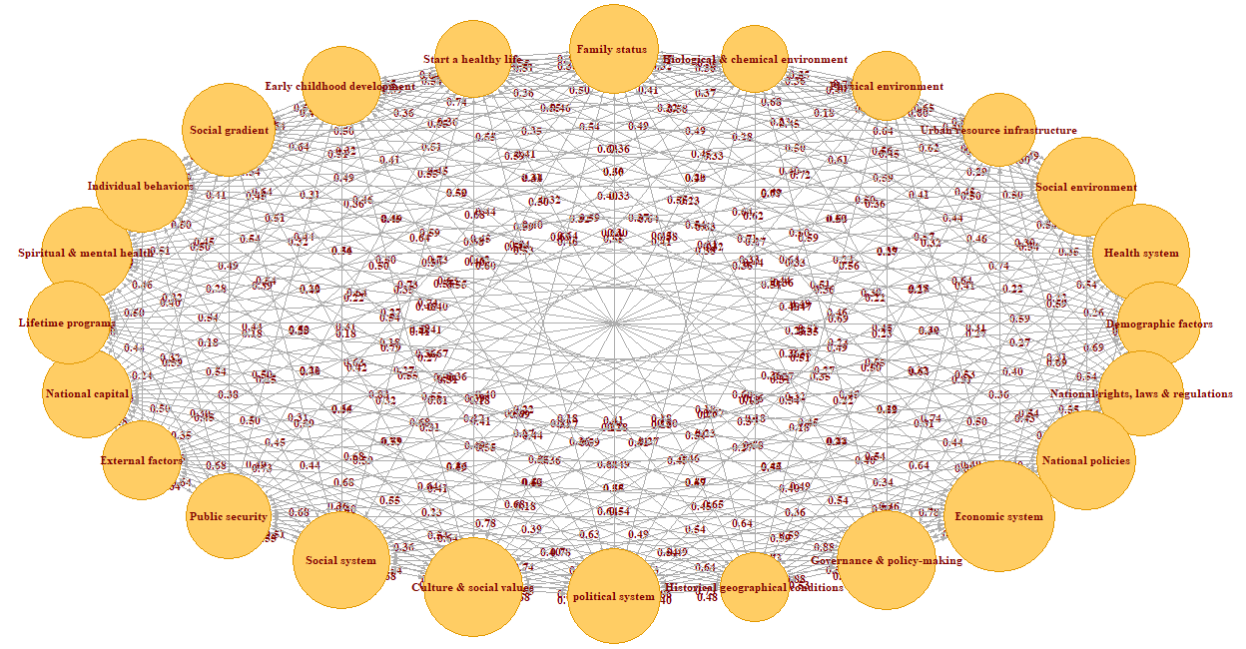
Fuzzy cognitive map of social determinants of health in Iran-internal factors

 This research considers health as a comprehensive factor influenced by various factors at the micro and macro levels to draw two comprehensive cognitive maps of the determinants of population health in Iran via two approaches: (1) only considering internal factors and (2) considering both internal and external factors simultaneously. The cognitive map in Figure 2 depicts the importance of internal factors affecting the population health and their casual relationships. According to this map, the most important social determinants of health are the economic system, culture and social values, governance and policy-making, national policies, and social environment with DoC 28.01, 25.18, 25.01, 24.97, and 24.88, respectively (Table 1). Analyzing these factors can improve the whole system and the population health. Among them, the most effective factors based on the od index are the economic system, governance and policy-making, national policies, culture and social values, and political system. Moreover, the most impressible factors in determining health based on the id index are the social environment, economic system, health system, spiritual and mental health, and individual behaviors.

**Table 1:** Degree of the centrality of social determinants of health and internal factors

**Levels**	**Out-degree**	**In-degree**	**Centrality**
Individual level			
Lifetime programs	9.61	11.49	21.10
Spiritual and mental health	10.86	12.28	23.14
Individual behaviors	11.39	12.14	23.53
Social gradient	11.66	11.87	23.52
Early childhood development	8.20	11.65	19.85
Start a healthy life	7.23	12.03	19.26
Family status	10.55	11.77	22.31
Local Level			
Biological and chemical environment	7.45	9.68	17.13
Physical environment	7.80	9.66	17.46
Urban resource infrastructure	8.86	9.40	18.26
Social environment	12.06	12.82	24.88
National level			
Health system	12.08	12.38	24.46
Demographic factors	10.43	10.59	21.02
National rights, laws and regulations	11.64	9.94	21.58
National policies	14.05	10.92	24.97
Economic system	15.45	12.56	28.01
Governance and policy-making	14.34	10.67	25.01
Historical geographical conditions	8.50	8.78	17.28
Political system	13.40	10.35	23.75
Culture and social values	13.73	11.44	25.18
Social system	12.80	11.77	24.56
Public security	10.84	10.87	21.70
External factors	11.13	8.79	19.92
National capital	11.01	11.25	22.26

**Figure 3:**
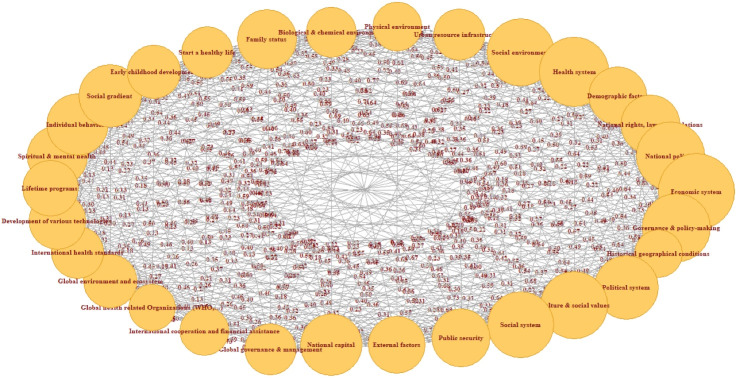
Fuzzy cognitive map of social determinants of health in Iran-internal and external factors

Figure 3 expands the scope of analysis by considering both the internal and external factors affecting health to show the FCM of the social determinants of health. Among all the internal and external factors affecting health, the economic system (33.27) is still the most important, followed by the health system (30.73), governance and policy-making (30.15), national policies (30.11), and culture and social values (29.58) (Table 2). Therefore, the economic system is still the most effective factor in this case. Among the social determinants of health in this model, the health system with an id of 15.90 is most affected by other factors.

**Table 2:** Degree of the centrality of social determinants of health-internal and external factors

**Levels**	**Out-degree**	**In-degree**	**Centrality**
Individual level			
Lifetime programs	10.68	13.79	24.48
Spiritual and mental health	12.21	14.43	26.63
Individual behaviors	12.59	14.64	27.24
Social gradient	13.45	14.27	27.72
Early childhood development	9.00	14.33	23.33
Start a healthy life	8.03	14.53	22.56
Family status	11.89	14.23	26.12
Local level			
Biological and chemical environment	9.66	12.44	22.10
Physical environment	9.66	11.85	21.51
Urban resource infrastructure	10.78	11.73	22.52
Social environment	14.16	15.33	29.49
National level			
Health system	14.83	15.90	30.73
Demographic factors	12.42	12.88	25.30
National rights, laws and regulations	14.21	12.00	26.21
National policies	16.93	13.18	30.11
Economic system	18.24	15.02	33.27
Governance and policy-making	17.13	13.02	30.15
Historical geographical conditions	10.20	10.66	20.86
Political system	15.78	12.39	28.17
Culture and social values	15.97	13.61	29.58
Social system	15.02	13.98	29.00
Public security	12.39	12.89	25.28
External factors	13.98	11.17	25.14
National capital	13.55	13.49	27.04
Global governance and management	12.19	10.28	22.47
International cooperation and financial assistance	11.13	10.42	21.55
Global Health-related Organizations (e.g., WHO)	10.35	10.11	20.46
Global environment and ecosystem	11.77	11.93	23.70
International health standards	12.68	10.37	23.04
Development of various technologies	14.48	10.52	25.00

## Discussion

 The current study aimed to identify the most important determinants of health in Iran and determine the cause-and-effect relationships among these factors using FCMs. The FCMs are a computational intelligence modeling and inference methodology suitable for modeling complex processes and systems of many highly-related and interconnected elements and subsystems.^21^ This FCM’s applicability to the model complex system has been successfully used in various application areas.^19-22,26,28-32^

 The health system and its social determinants are also known as a complex system due to a large number of factors and complex cause-and-effect relationships between these factors. Therefore, most of the studies conducted in the field of mapping of the social determinants of health have been performed qualitatively.^33-36^ Additionally, due to the difficulty of analyzing a large number of factors in a complex system, most quantitative studies only focus on cause-and-effect relationships with an emphasis on a specific factor such as lifestyle,^37^ health communication and media,^38^ immigration policies,^6^ human environment and habitat,^39^ globalization,^40^ focusing on social relations,^41^ and conflict.^42^

 The FCMs can clearly show which concepts influence other concepts and what this degree of influence is. These maps can represent cyclic dynamics^22^ when a factor is both a cause and an effect of another or when a self-pointing arrow indicates reinforcing internal dynamic.^20^ Consequently, the use of FCMs for analyzing complex health systems can be useful. The analysis of the FCMs show three values of the DoC, od, and id for all factors. These values determine the importance (based on centrality degree) and the effect of health determinants on each other. The findings of this research show that the most important social determinants of health in Iran are economic system, culture and social values, governance and policy-making, national policies and social environment in internal factors and economic system, health system, governance and policy-making, national policies and culture and social values in internal and external factors. Therefore, health in Iran largely depends on factors outside the health sector. In addition, Ramezani et al^43^ showed that factors beyond the health sector could considerably explain most of the health inequalities in Shiraz. Specifically, the present study shows that the economic system plays an important role in population health in Iran, which is in line with the previous studies. As an advantage compared with the previous studies, this study uses a qualitative methodology and covers the causal relationships.^36,44^

 The present research has the following practical recommendations for policy-makers.

- Health is a comprehensive subject strongly influenced by components outside the health system. Therefore, policy-makers should consider health as a comprehensive issue and inform different sectors about the results of their performance on health. Also, decision-makers should consider the positive and negative effects of each program on health when developing policies and programs.
- Considering the impact of the other sectors on health, especially macro factors such as economic conditions and national policies, decision-makers should consider inter-sectoral relations and develop concrete mechanisms to establish effective coordination in the government.
- The government should support synergies between and within different parts of the government in order to achieve a healthy society policy and provide the health sector with the possibility of having leadership power within the government.
- Considering the current limitations of conducting research of this kind, policy-makers should further have close cooperation with researchers to develop the knowledge base and methodology to understand the health determinants and the ways by which such factors are affected by public policies at all levels. These factors include the evaluation of current effects and relationships between public policies and subsequent evaluations.

In addition, this paper has the following theoretical suggestions for other researchers.

- Regarding the most important determinants of social health identified in this research, other researchers should evaluate the status of the determinants of the population’s social health in the model presented in this study. This evaluation shows the gap between the desired status and the existing status, and identifies the desired priorities by combining the importance of each component in the identified gap to improve the situation.

As one of the main applications of the FCM is in the field of scenario writing, researchers should use the maps prepared in this study for future research on population health in order to develop future scenarios.

 Determining cause-and-effect relationships by experts can be one of the limitations of the present study. The finding may have been affected by some errors in data collection, registration, and reporting, which cloud not be detected despite quality control efforts.

 An additional concern is that weighting the strength of relationships on the maps increases the length of the mapping sessions considerably, which risks reducing participant engagement. This challenge is more significant when multiple participants build the maps. In some concepts of causality, an outcome is the result of all interactions across the whole system.

## Conclusion

 By representing the causal relationships and determining the most important, effective, and impressible factors, the FCM of social determinants of public health helps the policy makers to understand the priorities and the links among the sectors to design, plan, and implement the health-oriented policies in all the sectors. This approach also evaluates the effects of current policies by creating links among policies and interventions as well as health determinants, consequences, and outcomes aiming at informing policy makers.

 Among the health determinants, the economic system has been one of the most important and the most effective factors in determining the population health in Iran in the last decade. Inflationary pressures, high unemployment rate, poverty, and unfair distribution of income have had severe effects on people’s lives and health in recent years. In addition, international sanctions have intensively affected the economic and livelihood situation and aggravated the effects of the economic system on the public health. Furthermore, the importance of the health system has changed by including the global factors in the analysis. The inclusion of the global factors, e.g., global technologies, international communication, and global organizations such as WHO, has increased in the impressibility of the health system, placing it among the first five components in terms of importance.

**Highlights**

- The economic system, health system, governance and policy-making are the most important factors among the social determinants of health in Iran.
- Economic conditions are the most critical social determinants of health in Iran.
- International sanctions are a crucial health determinant in Iran in recent years.
- In Iran, economic policies are more effective than health system policies in health status.

## Conflicts of interest

 The authors declare that there were no conflicts of interest in this study.

## Funding

 There was no funding for this study.
